# Numerical Modeling of Silicon Photodiodes for High-Accuracy Applications Part I. Simulation Programs

**DOI:** 10.6028/jres.096.023

**Published:** 1991

**Authors:** Jon Geist, Deane Chandler-Horowitz, A. M. Robinson, C. R. James

**Affiliations:** National Institute of Standards and Technology, Gaithersburg, MD 20899; University of Alberta, Alberta, Canada T6G 2G7

**Keywords:** high accuracy, internal quantum efficiency, PC-1D, photodiode modeling, silicon photodiodes

## Abstract

The suitability of the semiconductor-device modeling program PC-1D for high-accuracy simulation of silicon photodiodes is discussed. A set of user interface programs optimized to support high-accuracy batch-mode operation of PC-1D for modeling the internal quantum efficiency of photodiodes is also described. The optimization includes correction for the dark current under reverse- and forward-bias conditions before calculating the quantum efficiency, and easy access to the highest numerical accuracy available from PC-1D, neither of which is conveniently available with PC-1D’s standard user interface.

## 1. Introduction

PC-1D^1^ [1,2] is a computer program for numerical modeling of the electrical performance of one-dimensional semiconductor devices including photogeneration. It is optimized for solar cell modeling, and runs on IBM PC-compatible computers equipped with a numerical coprocessor. Version 2 of PC-1D includes realistic models of most of the semiconductor material and device properties that are important for high-accuracy applications of photodiodes. Consequently, Version 2 of PC-1D has the potential to be a tool for supporting these applications.

Unfortunately, for reasons that have to do with the difference between the applications for high-accuracy photodiodes and those for solar cells, it is not easy to achieve the highest accuracy of PC-1D through its standard user interface. Luckily, the designer of Version 2 of PC-1D anticipated this sort of problem and included the option to run it in batch mode in a way that does allow access to the highest levels of numerical precision and accuracy that are available from PC-1D.

This paper reports the development of a program shell for PC-1D that provides a batch-mode user interface optimized for high-accuracy modeling of photodiodes. The shell consists of three programs. The first is a program that prepares the input for PC-1D, the second is a program that reads the output from PC-1D, and the third is a MS-DOS batch file program that supervises the sequential execution of PC-1D and the other two programs.

The remainder of Part I of this series of papers reviews PC-1D and describes the new program shell for high-accuracy modeling of photodiodes. Typical applications are described in Parts II and III of this series of papers.

## 2. Description of PC-1D

PC-1D is an interactive, graphical, semiconductor-device simulation program that solves the fully-coupled, drift-diffusion (electron and hole transport) equations in one dimension. Its principal limitations are that it supports no more than three regions of possibly different materials, and that it is a one-dimensional model with a maximum of 150 finite elements. Within these constraints, the program is very versatile. It is well beyond the scope of this paper to describe its various capabilities.

Both the IBM Pascal source code and executable code are available. The latter requires an IBM PC-compatible computer equipped with at least 512 Kbytes of random access memory (RAM), a 80×87 coprocessor chip, a CGA, VGA, or EGA compatible graphics adapter, and a matching display. To use the shell described in this paper, batch-mode operation is necessary which requires an MS-DOS compatible operating system (Version 3.1 or greater).

Only one of the interactive modes of PC-1D is of interest for the purposes of this paper, the one that allows the creation and saving of parameter files. Parameter files contain all of the information needed by PC-1D to model a specific, user-defined device. These files are stored in binary format under user-defined names with .PRM as their extension. Included in the parameter file are the names of other data files needed by PC-1D to model the device.

### 2.1 Opening Menu

**Figure 1:**
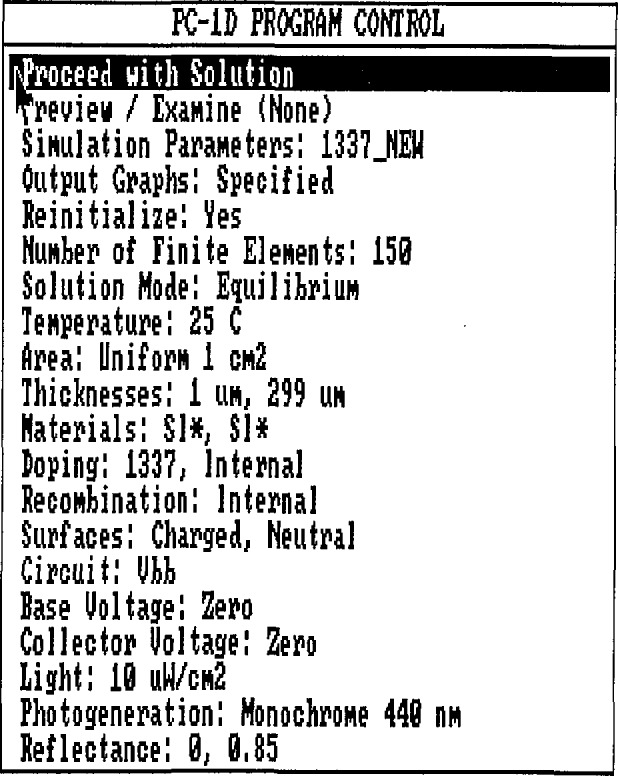
The opening menu of PC-1D when run in the interactive mode.

Figure 1 shows the opening menu when PC-1D is run in the interactive mode. An overview of the options mentioned in this menu is appropriate for what follows. The first, second, and fourth options, “Proceed with Solution,” “Preview / Examine,” and “Output Graphs:” are used in the interactive modeling mode, and are of no concern here. The “Solution Mode:,” “Base Voltage:,” “Collector Voltage:,” and “Light:” options are used to set the electrical and optical conditions under which the simulation will be run. For the purposes of this paper, it does not matter what selections are made for these options, because they will be set to the correct values by the high-accuracy photodiode modeling shell when it is run.

The “Simulation Parameters:” option is used to load parameter files in the interactive mode, but it is also used to save parameter files, which is of interest to the use of PC-1D in batch mode. The “Reinitialize:” and “Number of Finite Elements:” options allow details of the numerical procedures to be modified to a certain extent. Normally, 150 finite elements, the maximum allowed, will be needed for high-accuracy modeling. It will sometimes be necessary to use this menu option in order to force PC-1D to use all 150 elements. Reinitialization is probably desirable except, perhaps, when simulating a reverse-bias experiment.

The remaining options define the detailed nature of the device and external circuit to be modeled. All of the parameters obtained from the internal models built into PC-1D are adjusted to the values appropriate for the temperature entered following the “Temperature:” heading at the bottom of the opening menu. The user, of course, must assure that any user-defined parameters or data files are consistent with the chosen temperature. The “Area:” option is straightforward. The “Thickness:” option allows three different regions to be defined, each having its own thickness, material, and doping properties. If the thickness of a region is set to zero, it is not included in the solution, and it is not explicitly indicated following the “Thickness:” heading.

#### 2.1.1 Materials Properties

Selection of the “Materials:” heading of the opening menu allows such properties as the carrier mobilities, band-gap narrowing, Auger recombination cross sections, and absorption coefficient to be adjusted. For each region, a set of three files having the extensions .MAT, .INR, and .ABS are needed. These files are 1) an ASCII file that is the main material file, 2) an ASCII file containing wavelength versus real index of refraction data, and 3) an ASCII file containing wavelength versus absorption-coefficient data, respectively. All of the file names are stored in the parameter (PRM) file. In default operation, the INR and ABS files will have the same name as the MAT file. The user has the option of associating alternate ABS and INR files with a given MAT file in a particular parameter file, even though they have different names. If this is done, asterisks follow the MAT file names in the “Materials:” option heading, as shown in figure 1. Other material properties are included in internal models. Some of these allow the user to specify the values of the parameters in the equations defining the models, and most can be replaced by user-supplied ASCII files having the appropriate extensions. The internal model of the absorption coefficient of silicon, which is discussed next, is an example.

The default absorption-coefficient data in the SLABS file of Version 2 of PC-1D are not accurate enough for high-accuracy modeling of silicon photodiodes over the 400 to 900 nm spectral region [3]. Therefore, two data sets of higher accuracy were calculated and stored in files named SILW_EAK.ABS and SIL_PHIL.ABS. The first data set was calculated by using eq (1) of reference [3], which was fitted to the data of reference [4] over the spectral range from 470 to 1180 nm. The second data set was calculated by interpolation of the data of reference [5]. The first set is expected to be more accurate at longer wavelengths, and the second at shorter wavelengths.

**Figure 2:**
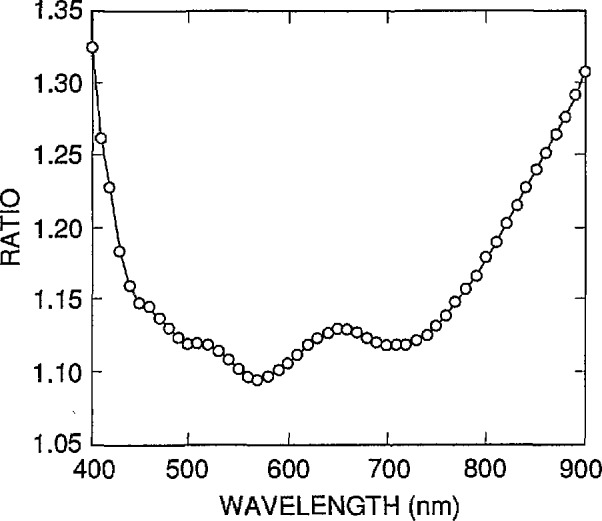
Ratios of the absorption-coefficient data interpolated from reference [5] to those calculated from eq (1) of reference [3] that were fitted to the data of reference [4].

The ratios of the data in SIL_PHIL.ABS to that in SIL_WEAK.ABS are plotted over the 400 to 900 nm spectral range in figure 2. The difference can be characterized as 12% ± 4% over the spectral region from 440 to 780 nm, growing much larger outside that spectral region. The value of 12% is a convenient average offset because it is the difference between the two data sets at 633 nm. At that wavelength, the value calculated from eq (1) of reference [3] agrees with a recent measurement [6] to within the ±2% uncertainty associated with the measurement.

One material property not modeled by PC-1D is the quantum yield for electron hole pair production [7]. This quantity may differ significantly from unity outside the 400 to 900 nm spectral region [8], but no high accuracy models currently exist [7,9]. This is the main reason that the modeling described in Part II of this series of papers is confined to the 400 to 900 nm spectral region. However, other problems, such as larger uncertainties in the available absorption-coefficient data at shorter wavelengths and uncertainties in the fraction of the radiation reflected by the rear surface of the photodiode at the longer wavelengths, also contribute to a significant deterioration in accuracy outside that spectral region.

#### 2.1.2 Device Properties

The “Doping:” heading of the opening menu of PC-1D allows either an internal doping model or an external file containing doping concentrations for each region of the device being modeled. The internal model consists of two front and two rear dopant distributions, as well as a uniform background dopant. The distributions can be chosen from uniform, complementary error, and Gaussian functions, and the parameters defining the maximum value, its location, and the width of the distributions can be adjusted. If tabular doping data are to be used, they are read from a user-generated ASCII file containing a depth, an *n*-type dopant concentration, and a *p*-type dopant concentration on each line, and having .DOP as its extension.

The shape of the equilibrium majority-carrier concentration near the front-surface oxide-silicon interface is very important to high-accuracy photodiode modeling [10]. It is necessary to use external files to model the front region doping in *p* ^+^*n*-type photodiodes to force PC-1D to devote enough finite elements to the front region to approximate accurately the majority-carrier concentration there.

The “Recombination:” heading of the opening menu of PC-1D allows the defect-related recombination in the volume and at the surfaces of the device to be modeled. (Auger recombination is considered a material property and is covered under the “Materials:” heading.) Shockley-Read-Hall (SRH) recombination through a single energy-level trap state is used as the model for volume recombination. Either an internal model or a user-defined table of depths and electron and hole lifetimes (equivalent to cross sections in the SRH model) in an external ASCII file with the extension .TAU can be used to model the volume recombination. With the internal model, the user may choose a single electron lifetime, a single hole lifetime, and a single trap energy relative to mid-gap for each region. With the external model, a trap level at mid-gap is used with the lifetime data in the external ASCII file.

**Figure 3:**
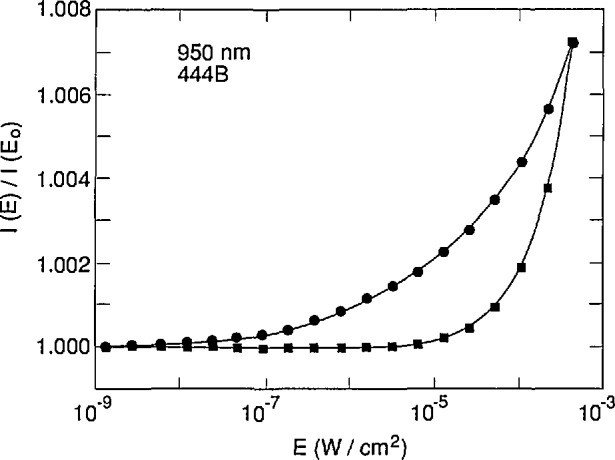
Comparison of experimental (filled circles) and simulated (filled squares) linearity measurements on an EG&G UV444B photodiode at 950 nm using a single SRH trap level at mid-gap with electron and hole lifetimes of 74.04 *µ*s.

**Figure 4:**
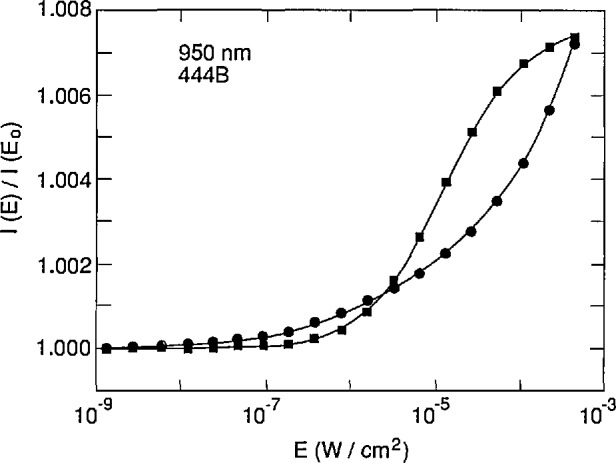
Comparison of experimental (filled circles) and simulated (filled squares) linearity measurements on an EG&G UV444B photodiode at 950 nm using a single SRH trap level 0.1796 eV above mid-gap with electron and hole (check for vice versa) lifetimes of 900 and 700 ms, respectively.

The restriction of the SRH model to a single-energy state prevents PC-1D from accurately fitting the measured [11] variation of quantum efficiency with flux (i.e., nonlinearity) at 950 nm for an EG&G UV444B photodiode as illustrated in figure 3. The simulated data in figure 3 were calculated using a mid-gap state with equal electron and hole lifetimes. Even though the simulated and experimental data agree in the low flux limit and again at a high flux level, their shapes are very different. Better agreement can be achieved at low flux levels at the cost of a worse fit at the higher flux levels by moving the state away from mid-gap, and by using different electron and hole lifetimes as illustrated in figure 4. It is expected that the proper distribution of SRH trap levels over the band gap would result in an accurate simulation of the nonlinearity of this type of photodiode.

It should be possible to develop an external lifetime model based on a parameterized distribution of SRH recombination states, and to adjust the parameters to fit the nonlinearity data shown in figures 3 and 4. However, it would be necessary alternately to run PC-1D to calculate the carrier concentrations and then the external model to calculate the lifetimes appropriate to these carrier concentrations, and to iterate to self-consistency. This is beyond the scope of the work reported in this paper.

The failure of the model to describe the nonlinearity of this type of photodiode casts some doubt on its ability to model accurately the reverse-bias self-calibration experiment [12–13]. This point is addressed in more detail in Part II of this series of papers.

In the case of surface recombination, the “Recombination:” option allows the choice of one of two internal models, a surface-recombination velocity model, and a saturation-current density model, but does not accommodate a user-defined data file. The choice of the surface-recombination velocity model allows the user to adjust the hole-and electron-recombination velocities as well as the energy-level of a single-energy surface state.

The “Surfaces:” option allows the user to choose the surface charge at the front and back surfaces of the device. For modeling the oxide-bias self-calibration experiment [12–13], the surface charge is the algebraic sum of any charge trapped in the oxide as a result of thermal oxidation and the charge stored on the oxide surface by the voltage applied to the transparent electrode.

The “Circuit:” option of the main menu of PC-1D allows the user to define a circuit in which the device is to operate. For the case of a photodiode, the emitter and base are connected, the collector is disconnected, and the connections to the device are made at the front and back surface. Keeping the collector disconnected forces the “Collector Voltage:” option to zero, as shown in figure 1. Setting the internal resistance *R_b_* to some non-zero value allows the effect of series resistance to be simulated.

## 3. The Photodiode Modeling Shell Programs

**Figure 5:**
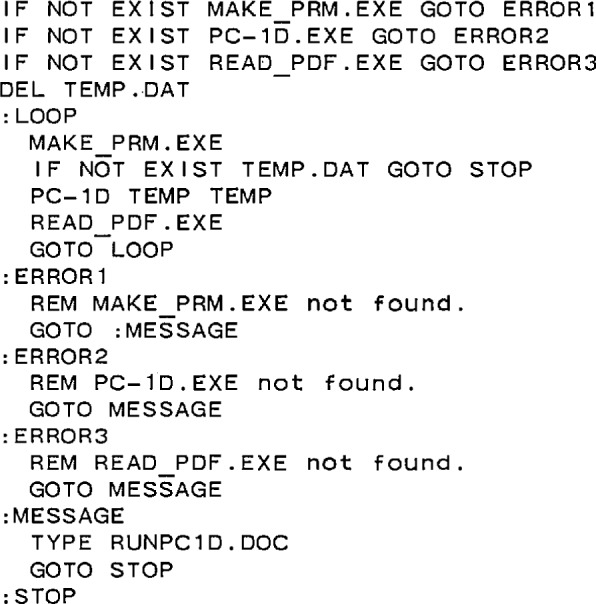
Listing for the MSDOS batch file RUN_C1D.BAT that supervises the execution of the programs MAKE_PRM.EXE, PC-1D.EXE, and READ_PDF.EXE for high-accuracy photodiode modeling.

The photodiode modeling shell for Version 2 of PC-1D is designed to allow simulation of oxide-bias and reverse-bias, self-calibration experiments, non-linearity measurements, and internal quantum efficiency spectra, while accessing the highest accuracy and precision available from PC-1D. It consists of three program files. The first program, a short MS-DOS Batch program named RUN_PC1D.BAT, is listed in figure 5. The other two programs, MAKE_PRM.EXE and READ_PDF.EXE, were complied with Version 5.5 of Turbo Pascal. Listings of the source code for these programs are given in reference [14].

### 3.1 Operation of RUN_PC1D.BAT

The first thing that RUN_PC1D.BAT does when run is to test whether or not the files MAKE_PRM.EXE, PC-1D.EXE, and READ_PDF.EXE all exist. If not, it issues an error message, and terminates execution at :STOP. If all three files do exist, RUN_PC1D deletes the temporary file TEMP.DAT. This is a precaution in case a previous nonstandard termination left this file in existence. RUN_PC1D then enters the loop between :LOOP and GO TO LOOP. Once in this loop, it transfers control to MAKE_PRM.EXE.

The first thing that MAKE_PRM.EXE does is to test the existence of TEMP.DAT. If TEMP.DAT does not exist, then MAKE_PRM.EXE prompts the user to define the photodiode experiment to be simulated. Examples of the questions and typical answers are shown in Appendix A. The questions and answers are straightforward and require no explanation. If the file option rather than the keyboard option is chosen to define the independent variables for the experiment to be simulated, then the program asks for the file name instead of the start, stop, and step values for the independent variable. Once MAKE_PRM.EXE has obtained all of the necessary information, it writes that information into the file TEMP.DAT, creates the temporary parameter file TEMP.PRM, and passes control back to RUN_PC1D.BAT.

RUN_PC1D.BAT now tests the existence of TEMP.DAT. If it does not exist, RUN_PC1D terminates execution. If TEMP.DAT does exist, then RUN_PC1D passes control to PC-1D.EXE. This program reads TEMP.PRM (the first TEMP on the command line following PC-1D), carries out the simulation defined therein, writes a table having a depth, an electron current, a hole current, and a total current on each line in the file TEMP.PDF (the second TEMP on the command line), and returns control to RUN_PC1D.BAT. Note that PDF files are the standard ASCII format HP plotter output files generated by PC-1D.

RUN_PC1D.BAT now passes control to READ_PDF.EXE. This program reads the total photocurrent at the depth specified in the definition of the experiment to be simulated (see Appendix A) from file TEMP.PDF, and appends it to the output file specified in response to the prompt from MAKE_PRM.EXE.READ_PDF.EXE then returns control to RUN_PC1D.BAT.

On subsequent passes through the loop, MAKE_PRM.EXE finds that TEMP.PRM does exist, reads the necessary information from this file, and writes updated TEMP.DAT and TEMP.PRM files. The information about when to exit the loop is in TEMP.DAT, and when MAKE_PRM.EXE determines that this time has come, it deletes TEMP.DAT, triggering RUN_PC1D.BAT to terminate execution.

### 3.2 Features of MAKEL_PRM and READ_PDF

A few points about MAKE_PRM.EXE and READ_PDF.EXE are in order. First, these two programs are designed to compensate for traits of PC-1D that make it less than ideal for high-accuracy modeling of photodiodes. The first of these is that PC-1D.EXE calculates the internal quantum efficiency from the sum of the photocurrent and the dark current occurring under reverse or forward bias. This may not be a problem in solar cell modeling, but it is not correct for photodiode modeling. As a result, MAKE_PRM.EXE calculates and stores the appropriate dark currents for all simulations that have a nonzero bias voltage *V_bb_*, and READ_PRM.EXE reads them and subtracts them from the appropriate total currents when calculating the quantum efficiency. This is simulated as a shutter-closed/shutter-open measurement sequence by MAKE_PRM.EXE.

**Figure 6:**
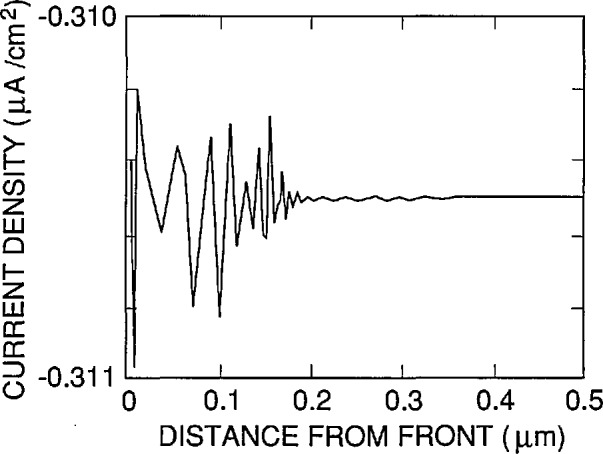
Variation in the total photocurrent calculated by PC-1D as a function of position in the front region of a Hamamatsu 1337 type photodiode due to the effect of the high front region doping concentration.

Even though the total current is a constant throughout a photodiode, it must be calculated at each finite element in the photodiode. The numerical precision with which it can be calculated depends upon the majority-carrier concentration in the element where it is being calculated due to the nature of the numerical algorithm, which involves the difference of two large numbers that scale with doping concentration. This is illustrated in figure 6, which shows the variation in the total current computed by PC-1D as a function of depth in the front region of a *p+n* -type photodiode irradiated by 1 *µ*W/cm^2^ of 440 nm radiation. This result was obtained by running PC-1D through its standard user interface and could not be obtained by running PC-1D with the shell just described, since carrier concentration as a function of depth is not an output supported by the shell. The decrease in the noise in the data with increasing distance from the front surface of the photodiode is evident in the figure.

PC-1D.EXE calculates the quantum efficiency from the total current at either the front or the rear of the photodiode at the discretion of the user. Since these are usually the regions where the majority-carrier concentration is greatest, PC-1D often calculates the quantum efficiency from values of the total photocurrent that are not the most precise available to it. This loss of precision, which occurs even in the absence of dark current, is the reason that MAKE_PRM.EXE asks the user to choose the depth at which the total photocurrent will be read from the TEMP.PDF file. This depth should be somewhere in the interior of the photodiode where the doping concentrations are low and the effect of surface fields on the carrier concentrations is small or negligible.

Very high precision is available under these conditions. The internal quantum efficiency calculated by RUN_PC1D for the same type of photodiode used to generate the data in figure 6, but with all loss (recombination) mechanisms set to zero, is 6 parts per million (ppm) above unity ±1 ppm for 1 *µ*W/cm^2^ irradiation anywhere within 400 to 900 nm spectral region. This result was obtained by assuming the somewhat large thickness of 400 mm for this photodiode to minimize the fraction of the incident radiation penetrating to the rear of the photodiode. Since PC-1D does not permit a reflectance of 100% at the rear surface of the diode, a more typical 300 mm thick photodiode would show a loss of a few ppm at the longest wavelengths.

Since PC-1D does not model the oxide passivation on the front surface of the photodiode, it is necessary to vary the oxide trapped charge N*_ss_* to simulate the effect of applying a voltage across the oxide in the silicon self-calibration experiment. First, the value of N*_ss_* must be chosen to simulate the oxide-fixed charge. To this value must be added the charge appropriate to the application of oxide bias according to the formula for the charge density stored on a capacitor as a function of thickness, dielectric constant, and applied voltage. It is this sum that PC-1D must use as N*_ss_* when simulating the effect of oxide bias on a photodiode. The bookkeeping for the values of N*_ss_* chosen to simulate the oxide-fixed charge and the values of N*_ss_* needed to simulate the combined effect of the fixed charge and the applied oxide bias are handled by MAKE_PRM.EXE.

## 4. Conclusion

The semiconductor device modeling program PC-1D and some programs to enhance its use in the high-accuracy modeling of silicon photodiodes have been described. The features of PC-1D that suit it for high-accuracy photodiode modeling have been described, and the features of the support programs that compensate for features of the PC-1D user interface that are less than ideal for this application have also been described. Examples of the use of these programs in photodiode modeling in different types of high-accuracy applications are given in Parts II and III of this series of papers.

## Footnotes

1 Certain commercial equipment, instruments, or materials are identified in this paper to specify adequately the experimental procedure. Such identification does not imply recommendation or endorsement by the National Institute of Standard and Technology, nor does it imply that the materials or equipment identified are necessarily the best available for the purpose.

## 5. Appendix A

A Sample of the Screen Produced by MAKE_PRM.EXE When RUN_PC1D.BAT is Executed.

Parameter file (including PRM extension) defining photodiode:

1337_new.prm

Complete path to file in which to store QE results:

test.dat

Type of experiment to simulate?

(O)xide bias (Volts) scan?

(R)everse bias (Volts) scan?

(W)avelength (nm) scan?

(P)hoton energy (eV) scan?

(L)inearity (W/cm^^^2) scan?

Enter letter here: w

Start, stop, and stop values from (K)eyboard or all

values from (F)ile? k

First value for scanned parameter: 400

Stop value for scanned parameter: 900

Step size for scanned parameter: 10

Incident irradiance (W per sq. cm): 1e-5

Oxide bias voltage (V): 0

Reverse bias voltage (V): 0

Non-zero thickness of front surface oxide (nm): 25

Depth at which to get total photocurrent (um): 100
